# ACE2, Much More Than Just a Receptor for SARS-COV-2

**DOI:** 10.3389/fcimb.2020.00317

**Published:** 2020-06-05

**Authors:** Lobelia Samavati, Bruce D. Uhal

**Affiliations:** ^1^Division of Pulmonary, Critical Care and Sleep Medicine, Department of Internal Medicine, School of Medicine and Detroit Medical Center, Wayne State University, Detroit, MI, United States; ^2^Center for Molecular Medicine and Genetics, Wayne State University School of Medicine, Detroit, MI, United States; ^3^Department of Physiology, Michigan State University, East Lansing, MI, United States

**Keywords:** COVID-19, lung, alveolar, angiotensin, coagulopathy

## Abstract

The rapidly evolving pandemic of severe acute respiratory syndrome coronavirus (SARS-CoV-2) infection worldwide cost many lives. The angiotensin converting enzyme-2 (ACE-2) has been identified as the receptor for the SARS-CoV-2 viral entry. As such, it is now receiving renewed attention as a potential target for anti-viral therapeutics. We review the physiological functions of ACE2 in the cardiovascular system and the lungs, and how the activation of ACE2/MAS/G protein coupled receptor contributes in reducing acute injury and inhibiting fibrogenesis of the lungs and protecting the cardiovascular system. In this perspective, we predominantly focus on the impact of SARS-CoV-2 infection on ACE2 and dysregulation of the protective effect of ACE2/MAS/G protein pathway vs. the deleterious effect of Renin/Angiotensin/Aldosterone. We discuss the potential effect of invasion of SARS-CoV-2 on the function of ACE2 and the loss of the protective effect of the ACE2/MAS pathway in alveolar epithelial cells and how this may amplify systemic deleterious effect of renin-angiotensin aldosterone system (RAS) in the host. Furthermore, we speculate the potential of exploiting the modulation of ACE2/MAS pathway as a natural protection of lung injury by modulation of ACE2/MAS axis or by developing targeted drugs to inhibit proteases required for viral entry.

## Introduction

Severe acute respiratory syndrome virus 2 (SARS-CoV-2) causes a respiratory disease that led to the fatal Coronavirus disease 2019 (COVID-19) pandemic. In late 2019, the SARS-CoV-2 outbreak was first reported in Wuhan, China that later led to a true crisis worldwide (Huang et al., 2020). Coronaviruses (CoVs) are large enveloped non-segmented positive-sense RNA viruses. They generally cause mild enteric and respiratory diseases in animals and humans (Glass et al., 2004). Most human CoVs, such as hCoV-229E, OC43, NL63, and HKU1 usually cause only mild respiratory diseases (Fouchier et al., 2004). SARS-CoV-2 causes acute, highly lethal pneumonia with clinical symptoms similar to those reported for SARS-CoV and MERS-CoV-2 (Fouchier et al., 2004). In contrast to SARS-CoV, SARS-CoV-2-infected patients rarely show prominent upper respiratory tract signs and symptoms. On presentation, most infected individuals exhibit dry cough (83–99%), and dyspnea (59.4–82%) with findings of bilateral ground-glass opacities on radiographic images (Guo et al., 2020; Huang et al., 2020). In most severe cases the characteristic symptom is respiratory distress (~55%) Grasselli et al.

The reported mortality varies based on race, sex, age, and comorbid conditions (Baud et al.; Porcheddu et al., 2020). Currently the true mortality still is not well-established, as the mortality may occur up to 30 days post infection. Based on current literature, most severe SARS-CoV-2 cases progressed within 14–21 days after disease onset. Various laboratory abnormalities have been observed even preceding the significant respiratory dysfunction (Lu et al., 2020). Mortality related to SARS-CoV-2 in China as reported by the WHO is about 3.4% (Guo et al., 2020; Sohrabi et al., 2020). The most severe cases have been predominantly reported in elderly or subjects with preexisting conditions, predominantly cardiovascular diseases such as hypertension and congestive heart failure (Zhou et al., 2020). Interestingly, these risk factors are similar to the reported risk factors (diabetes, hypertension, obesity) associated with MERS-CoV related mortality, although MERS-CoV respiratory disease occurred in younger individuals (Assiri et al., 2013; World Health Organization, 2013). These clinical and epidemiological observations may provide some direction on the mechanism of disease. Recent reports indicate that a significant portion of SARS-CoV-2 related hospitalization in the USA are below the age of 50 years. Given the fact of a higher prevalence of metabolic diseases, including obesity, hypertension, cardiovascular diseases and diabetes in the US population (Moore et al., 2017), this infection may cause higher mortality. The virus gains entrance into its host cell via the ACE2 receptor. How the known epidemiological and clinical manifestation of SARS-CoV-2 infection may be explained by perturbations of physiological functions of the ACE2 receptor due to receptor virus interaction will be discussed in this manuscript.

## SARS-CoV-2 Host Interaction

SARS-CoV-2 is single-stranded positive-sense RNA virus, containing ~26–32 kilobase (kb) genome. The viral envelope consists of a lipid bilayer, where the viral membrane (M), envelope (E), and spike (S) structural proteins are anchored. Unlike other corona viruses, SARS-CoV-2 does not use aminopeptidase N (APN) and dipeptidyl peptidase 4 (DPP4) as a receptor (Raj et al., 2013). Similar to SARS-CoV, SARS-CoV-2 utilizes a novel metallocarboxyl peptidase angiotensin receptor (ACE) 2 to gain entry into human cells (Donoghue et al., 2000; Turner et al., 2002; Li et al., 2003). Similar to other CoV, during viral entry into the host cell, the spike proteins (S) on the envelope of SARS-CoV-2 are cleaved into S1 and S2 subunits (Kirchdoerfer et al., 2016). S2 does not interact with the receptor but it harbors the functional elements required for membrane fusion of the virion. The S1 protein/receptor interaction is the pivotal determinant for SARS-CoV-2 to infect a host species. S1 contains the receptor binding domain (RBD) and directly binds to the peptidase domain (PD) of ACE 2 to gain entry into host cells (Turner et al., 2002; Li et al., 2003; Yan et al., 2020). Despite high similarity between the RBD of SARS-CoV and SARS-CoV-2, several amino acid variations are observed in the middle of the binding domain of SARS-CoV-2, which provide an increased affinity to bind to ACE2 more effectively (Wang Q. et al., 2020; Yan et al., 2020). Peptidase activity of ACE2 is critical for the virion to gain access into the host cytosol. Similar to SARS-CoV, proteolytic cleavage of S1 containing the receptor binding domain (RBD) at the C-terminus of S1 protein of SARS-CoV-2 is required to initiate interaction with PD of the ACE2 receptor (Li et al., 2005; Yan et al., 2020). Cleavage of S1 protein is achieved by acid-dependent proteolytic cleavage by one or several host proteases, including cathepsins, transmembrane protease serine protease (TMPRSS)2, TMPRSS4, or human airway trypsin-like protease (Hoffmann et al., 2020). The exact protease has not been identified. Proteolytic cleavage is followed by fusion of the viral and cellular membranes. Furthermore, it has been shown that S protein cleavage occurs at two different sites within the S2 portion of the protein, with the first cleavage important for separating the RBD and fusion domains of the S protein and the second for exposing the fusion peptide (cleavage at S2′) (Belouzard et al., 2009). Binding of S1 to the ACE2 receptor triggers the cleavage of ACE2 by a disintegrin and metallopeptidase domain 17 (ADAM17)/tumor necrosis factor-converting enzyme (TACE) at the ectodomain sites (Lambert et al., 2005; Heurich et al., 2014; Oarhe et al., 2015). Additionally, TMPRSS2 cleaves ACE2 at the intracellular C-terminal domain (Heurich et al., 2014; Hoffmann et al., 2020). Both cleavages (ectodomain and endodomain) by ADAM17 and TMPRSS2 facilitate effective cellular viral entry. It appears that this process leads to shedding of host ACE2 receptor (Belouzard et al., 2009) that may contribute to the loss of ACE2 function and systemic release of S1/ACE2 complex.

Generally fusion with the host plasma membrane occurs within acidified endosomes that requires cleavage at S2′ exposing a fusion peptide that inserts into the membrane. The potential beneficial effect of chloroquine on SARS-CoV-2 is due to its effect on the endosomal uptake and acidification. The process of fusion with the host membrane is followed by the formation of a funnel like structure built by two heptad repeats in the S2 protein in an antiparallel six-helix bundle facilitating the fusion and release of the viral genome into the cytoplasm. The viral replication genome of CoVs contains a variable number (World Health Organization, 2013; Lu et al., 2020; Porcheddu et al., 2020; Sohrabi et al., 2020; Zhou et al., 2020; Baud et al.) of open reading frames (ORFs). Two-thirds of viral RNA, mainly located in the first ORF (ORF1a/b) translates two polyproteins, pp1a and pp1ab, and this encodes 16 non-structural proteins (NSP), while the remaining ORFs encode accessory and structural proteins (Fehr and Perlman, 2015). The rest of the virus genome encodes four essential structural proteins, including spike (S) glycoprotein, small envelope (E) protein, matrix (M) protein, and nucleocapsid (N) protein (Fehr and Perlman, 2015). After replication and subgenomic RNA synthesis, the viral structural proteins, S, E, and M are translated and inserted into the endoplasmic reticulum (ER), followed by movement along the secretory pathway into the endoplasmic reticulum-Golgi intermediate (Krijnse-Locker et al., 1994; Fehr and Perlman, 2015). The M protein directs most protein-protein interactions. For assembly of virus, the interaction of M protein with E protein is required to form Virus-Like Particles (VLPs), suggesting these two proteins function together to produce coronavirus envelopes.

## Mechanism of Disease

### Effect of SARS-CoV-2 Infection on Renin/Angiotensin System

Because of the central role of ACE2 receptor as the viral entry point, the understanding of the functional role of ACE/angiotensin receptor (AT) and ACE2/MAS receptor is critical for the understanding of the pathophysiological changes due to SARS-CoV-2 infection. Understanding of the molecular downstream effects of angiotensin (Ang) on cellular signaling may explain the observed clinical picture of severe respiratory distress, myocardial injury, renal failure, and increased mortality due to SARS-CoV-2 infection among the aging population and subjects with cardiovascular and metabolic diseases (Zhou et al., 2020; Zhu et al., 2020).

### ACE Genes

**Figure 1:**
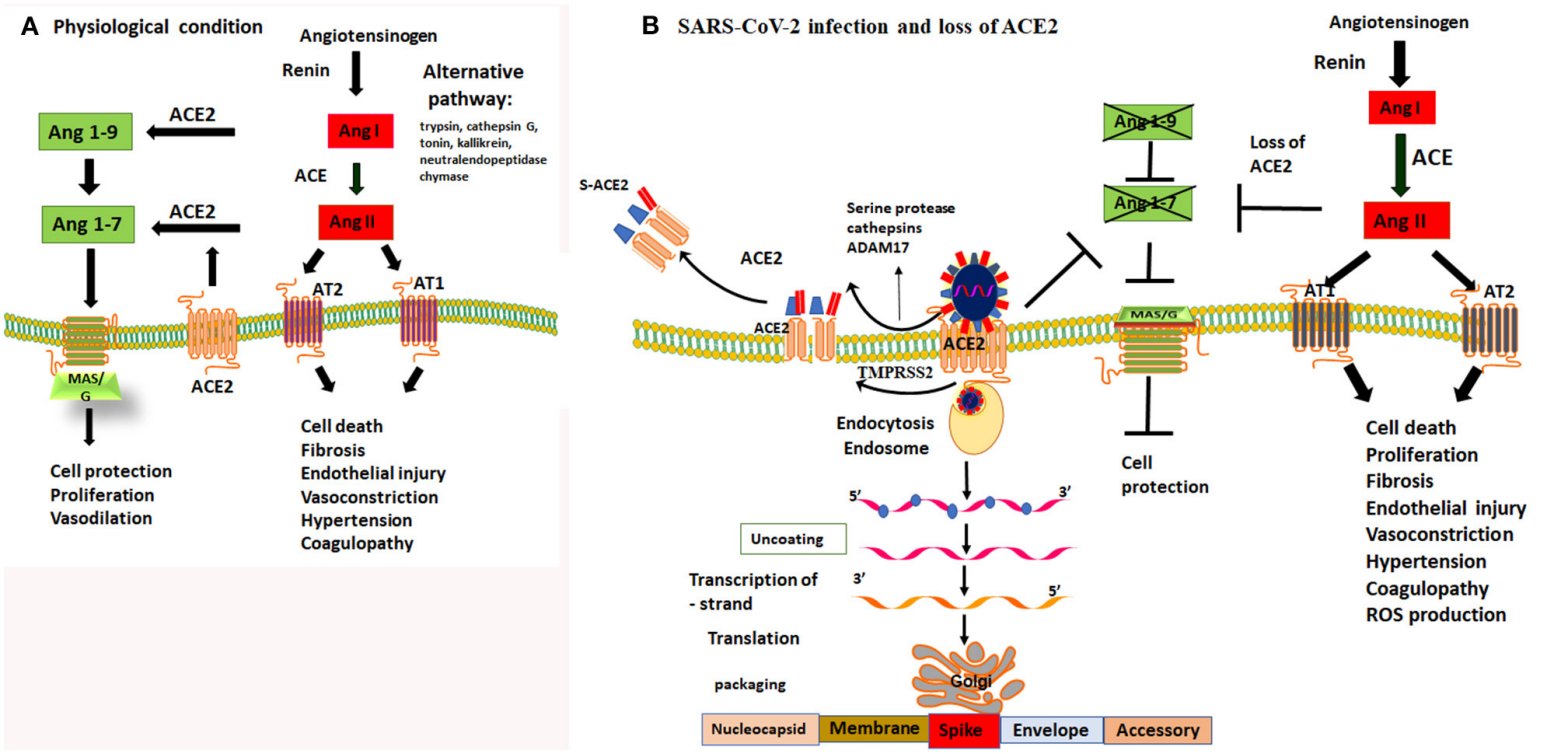
Dysregulation of Ang II and Ang (1-7) by loss of protective function of ACE2 receptor. **(A)** under physiological condition there is a balance in ACE and ACE2 receptor activity. ACE regulates the Renin Angiotensin Aldosterone system (RAS) and cleaves Ang I to produce Ang II. Ang II is a potent vasoconstrictor and detrimental for endothelial and epithelial function through activating AT1 and AT2 receptors. The counterbalance of the RAS/Ang II output is regulated by ACE2 and Mas/G protein coupled receptor activity. ACE2 cleaves Ang I and Ang II into Ang-1-9 and Ang1-7, respectively, thereby it activates MAS/G protein coupled receptor that protect cell death. **(B)** SARS-CoV-2 binds to ACE2 to gain entry to epithelial cells of the lungs. Cleavage of spike proteins by a protease such as trypsin/cathepsin G and or ADAM17 on ectodomain and TMPRSS2 of endodomain sites facilitate viral entry into the cells. This process leads to shedding of host ACE2 receptors and loss of its protective function. Loss of function of ACE2 activity prevents production of Ang 1-9 and Ang1-7. Lack of Ang1-7 diminishes the activity of MAS/G receptor, leading to the loss of its protective functions including vasodilatation, cell protection both at the epithelial and endothelial sites. Loss of ACE2 function leads to an imbalance and unchecked effects of Ang II and upregulation of RAS/Ang II pathway. Upregulation of Ang II leads to vasoconstriction, thrombophilia, microthrombosis, alveolar epithelial injury and respiratory failure. Therefore, inhibiting the proteolytic function of trypsin/cathepsin and ADAM17 or TMPRSS2 and or direct activation of MAS/G receptor by enhancing Ang-(1-7) can overcome the loss of function ACE2 and are viable targets to prevent tissue damage to the host.

Sequence analysis suggests that ACE and ACE2 exhibit 42% amino acid homology and ACE2 has evolved through gene duplication (Donoghue et al., 2000). ACE2 maps to chromosome Xp22, spans 39.98 kb of genomic DNA, and contains 20 introns and 18 exons (Turner et al., 2002). The ACE2 gene encodes a type I membrane-bound glycoprotein composed of 805 amino acids (Marian, 2013). Functional domains include a C-terminal transmembrane anchoring region (carboxy-terminal domain), N-terminal signal peptide region and an HEXXH zinc binding metalloprotease motif (catalytic domain) (Li et al., 2003; Cerdà-Costa and Xavier Gomis-Rüth, 2014). ACE receptors are expressed in almost all tissues, while ACE2 is expressed on alveolar epithelial cells and capillary endothelial cells. ACE2 is highly expressed in capillary rich organs such as lungs and kidneys but also in the gut and brain (Hamming et al., 2004; Tikellis and Thomas, 2012; Roca-Ho et al., 2017). Genetic polymorphisms of ACE and ACE2 are associated with hypertension, cardiovascular disease, stroke, and diabetes (Crackower et al., 2002; Ramachandran et al., 2008; Jang and Kim, 2012; Fehr and Perlman, 2015). Despite the structural homology between ACE and ACE2, they have divergent physiological function. ACE regulates the Renin Angiotensin Aldesterone system (RAS). ACE2 counterbalances the deleterious effect of the ACE/RAS pathway through its downstream ACE2/Angiotensin (1-7)/MAS axis. The critical role of RAS has been shown in the pathogenesis of metabolic inflammatory diseases (de Kloet et al., 2010). Classical activation of angiotensin II depends on renin and ACE activity. Prorenin (a 46KD protein) is the inactive precursor of renin. Upon activation of the juxtaglomerular apparatus (JG) of the afferent arterioles of the kidneys, specialized proteases cleave prorenin to renin. Once renin is released into the blood, it cleaves angiotensinogen into angiotensin (Ang) I. Ang I is physiologically inactive, but acts as a precursor of Ang II. The conversion of Ang I to Ang II is catalyzed by ACE. ACE is expressed primarily in the vascular endothelium of the lungs and kidneys (Wakahara et al., 2007), but also on the epithelium of the lungs and upper respiratory system. After Ang I is converted to Ang II, it binds to angiotensin II type I (AT) and type II receptors in the kidney, adrenal cortex, arterioles, and the brain (Figure 1A). Ang II acts on the adrenal cortex to stimulate the release of aldosterone (Xue et al., 2011), leading to sodium and water retention. While the effects of Ang II are rapid, the effects of aldosterone are retarted due to slower effects on downstream targeted gene transcription. The overall physiological net effects of RAS activation is an increase in total body sodium, total body water, and increased vascular tone. Furthermore, the binding of Ang II to AT receptors results in vasoconstriction (Gustafsson and Holstein-Rathlou, 1999), endothelial injury (Watanabe et al., 2005), endovascular thrombosis (Tay and Lip, 2008) and increase blood volume. Increased Ang II is associated with hypertension and accelerated thrombosis in arterioles by activating the coagulation cascade (both thrombin and platelets) (Senchenkova et al., 2010; Singh and Karnik, 2016). Interestingly, the thrombogenic effects of AngII on the platelets was not reversible by application of aspirin (Jagroop and Mikhailidis, 2000). At the cellular level, angiotensin II induces various signaling pathways, including serine/threonine kinase, ERK, JNK/MAPK as well as PKC (Malhotra et al., 2001). Studies have shown that Ang II effectively induces IL-6 and TNF-α, possibly through serine tyrosine kinases, ERK/JNK MAPK activation, G protein coupled receptor activation or through interaction with mineralocorticoid receptors (Funakoshi et al., 1999; Han et al., 1999; Ruiz-Ortega et al., 2002; Luther et al., 2006). Ang II is a potent activator of nicotinamide adenine dinucleotide phosphate (NADPH) oxidase and hence an inducer of reactive oxygen species (ROS) production (Garrido and Griendling, 2009). Furthermore, Ang II activates neutrophils and macrophages flux to the affected tissues and inhibits the production of nitric oxide and hence promotes vascular injury (Kato et al., 1996; Nabah et al., 2004). These considerations provide new visions to develop targeted therapies, as Ang II functions as a pluripotent mediator to enhance cytokines (IL-6, TNFα, and others), oxidative injury by ROS, endothelial injury by inhibiting NO synthesis and vasoconstriction. Therefore, inhibition of only one of its targets for instance IL-6 may not provide significant therapeutic benefit in these patients. Currently, there is an ongoing clinical trial to study the effect of monoclonal antibodies against IL-6 receptor (ClinicalTrials.gov Identifier: NCT04317092).

It is very important to note, especially in the context of SARS-CoV-2 infection, that besides the classical RAS/ACE mediated Ang II formation, formation of Ang II can occur through alternative pathways by various proteases. These include tryptensin, cathepsin G, tonin, kallikrein, neutral endopeptidase, and chymase (Figure 1A). These proteases can cleave Ang I to form Ang II (Kramkowski et al., 2006; Lorenz, 2010; Becari et al., 2011; Uehara et al., 2013). Most of these proteases are localized in specific tissues (lungs, myocardium, arterioles, kidney, or brain) and are not sensitive to ACE inhibitors. Interestingly, targeted inhibition of ACE using ACE inhibitors, only decreased Ang II levels for a short period of time, and Ang II levels return to baseline 1 week after treatment with ACE inhibitors (Mento and Wilkes, 1987). Furthermore, it has been shown that application of ACE and Ang II receptor blocker (ARB) inhibitors in animal models leads to an increase in the expression of ACE2 (Ishiyama et al., 2004). Part of protective function of ACE and ARBs is considered to be due to upregulation of ACE2. Therefore, it is possible that upregulation of ACE2 may provide more available receptors for viral entry and hence a higher viral load associated with poor prognosis (Chu et al., 2004). This also suggests that in subjects, who are on ACE inhibitors, the activation of alternative pathways may play a significant role in the formation of Ang II (Diaz, 2020). Currently, a clinical trial is ongoing to assess the effect of ACE/ARB inhibitors (ClinicalTrials.gov Identifier: NCT04330300) on SARS-CoV-2 infection. If the alternative pathways in the formation of Ang II are important, it is highly unlikely that the ACE/ARB inhibitors play a role on the clinical course of SARS-CoV-2 infection.

ACE2 acts as a ligand through its recently identified MAS1 receptor, which is a G-protein–coupled receptor (Donoghue et al., 2000; Santos et al., 2003). ACE2 is a monocarboxypeptidase, which cleaves Ang I into a non-apeptide, Ang 1-9 and Ang II into a heptapeptide, Ang 1-7 (Santos et al., 2003; Marian, 2013). Both peptides have vasodilatory and antiproliferative and protective functions by activating the MAS/G receptor. The ACE2/Ang 1-7/MAS1 axis provides an endogenous counter-regulatory mechanism within the renin–angiotensin system (RAS) that balances the deleterious effects of the ACE/Ang II/AT1 receptor axis (Santos et al., 2003). Mice deficient in MAS1 or ACE2 receptors exhibit cardiac systolic dysfunction, increased blood pressure, myocardial interstitial fibrosis, endothelial dysfunction, and exhibit increased susceptibility to intravascular thrombosis, chronic kidney disease, metabolic abnormalities, and various other biological abnormalities that regulate the cardiovascular system (Yamamoto et al., 2006; Tikellis and Thomas, 2012). ACE2 activation prevents the deleterious effects of Ang II on the cells and organisms, such as cell death, fibrosis, angiogenesis, and thrombosis formation (Fraga-Silva et al., 2010; Tikellis and Thomas, 2012). Recent autopsy results on SARS-CoV-2 infected humans showed diffuse alveolar damage with massive capillary congestion accompanied by microthrombi in vascular beds but a paucity of inflammatory infiltrates (Menter et al., 2020). However, pathological examination on autopsies have not investigated if SARS-CoV-2 infection leads to total destruction of ACE2 receptors on the alveolar epithelial and endothelial cells. Interestingly, in an animal model of SARS-CoV, Oudit et al. found a marked decreased ACE2 expression in the heart of infected mice (Oudit et al., 2009). The key product of ACE2 activity is Ang-(1-7), which is considered a biologically active member of the RAS. By binding to MAS, it induces many beneficial actions, such as vasodilation, inhibition of cell growth, and protection from alveolar epithelial cell injury. In addition, it has antifibrotic, anti-thrombotic, and antiarrhythmogenic effects (le Tran and Forster, 1997; Schindler et al., 2007; Li et al., 2008). It has been shown that the ACE2-Ang-(1-7)-MAS axis has a protective effect on the brain and prevents ischemic stroke (Jiang et al., 2013).

### Direct Protective Actions of ACE2 on Lung Alveolar Epithelial Cells

In addition to its protective role in the cardiovascular system, ACE2 has a direct protective role in alveolar epithelial cells. In the lungs ACE2 has numerous physiological functions, most of which are protective against lung injury. Similar to the endothelial site, ACE2 degrades the octapeptide Ang II by removing a single amino acid from the C-terminal end of the peptide to generate the heptapeptide Ang1-7. Our laboratory and others have shown that ACE2 protects against lung injury by: (a) degrading Ang II, which is vasoconstrictive and proapoptotic for lung epithelial cells (Wang et al., 1999) and profibrotic (Li et al., 2008; Uhal et al., 2011), and (b) by producing the peptide Ang1-7, which inhibits the actions of Ang II through binding to the MAS receptor (Gopallawa and Uhal, 2014). In support of this protective role for ACE2, pharmaceutical preparations of recombinant ACE2, when administered to experimental animals, protect against lung cell death, inhibit acute lung injury and prevent lung fibrosis after chronic injury to the lungs (Li et al., 2008; Rey-Parra et al., 2012). As further evidence, the application of a specific competitive inhibitor of ACE2, DX600, to primary cultures of isolated ACEs increases the level of Ang II released into the serum-free culture medium by autocrine mechanisms, reduces the amount of released Ang1-7 and, importantly, induces apoptosis inhibitable by the AT1 receptor blocker (Menter et al., 2020). Thus, functional ACE2 normally expressed by alveolar epithelial cells can be viewed as a critical survival factor for these lung cells. In addition, the enzymatic product of ACE2, the Ang1-7, itself protects against lung cells death by antagonizing that actions of Ang II (le Tran and Forster, 1997). If Ang1-7 is applied to cultures of lung epithelial cells, it can prevent lung cell death in response to either Ang II or the ER stress inducer MG132 (Nguyen and Uhal, 2016). The Ang1-7 receptor MAS and the JNK-selective phosphatase MKP-2 appear to be critical in this protective action of Ang1-7 response, becauses iRNAs or antisense knockdowns of MAS or MKP-2 can eliminate the ability of Ang1-7 to prevent lung cell death (Gopallawa and Uhal, 2016). Indeed, Ang1-7 itself and congeners of the peptide, such as cyclic Ang1-7 (Gopallawa and Uhal, 2016), have already been shown to protect the lungs in preclinical models of acute lung injury (Simoes e Silva et al., 2013; Gopallawa and Uhal, 2014).

### Therapeutic Strategies for SARS-CoV-2 Infection

Currently, there are no targeted drugs specifically against SARS-CoV-2. Recent efforts have been put forward of drug repurposing by screening of various available antiviral agents with the aim to identify possible treatments. Among those, lopinavir, originally used for treatment of human immunodeficiency virus, was identified to have potential antiviral activity against SARS-CoV-2. Unfortunately, a randomized-controlled, open-label trial involving hospitalized adult patients with confirmed SARS-CoV-2 infection showed no benefit of lopanavir (Cao et al., 2020). Other studies suggested that remdesivir (GS5734) an inhibitor of RNA polymerase, originally developed to treat Ebola infections, has *in vitro* activity against multiple RNA viruses, including SARS-CoV-2 (Mulangu et al., 2019). Experimental data suggested that at micromolar concentration of remdesivir and chloroquine potentially blocked virus infection (Wang M. et al., 2020). Current clinical trials are ongoing to assess the efficacy of remdesivir treatment alone or in conjunction with chloroquine in SARS-CoV-2 infection. Because hydroxychloroquine and chloroquine are considered inhibitors of endosomal trafficking of SARS-CoV-2, these drugs are used as potential therapeutics. Both drugs are antimalarial drugs that are also used as antiinflammatory drugs in various autoimmune diseases, including rheumatoid arthritis, Lupus erythematosus, and respiratory diseases such as sarcoidosis (Martin et al., 2009; Talreja et al., 2019). Despite the high media coverage, currently, there are no randomized clinical trials to support their efficacy against SARS-CoV-2 infection. However, it is conceivable that their efficacy may vary in different stages of virion life cycle and virus interaction with the host. These drugs may be beneficial in early stages of the infection, when the virus requires endosomal uptake. In fact, during the preparation of this manuscript, several non-randomized clinical trials have suggested a lack of significant efficacy of antimalarial drugs in the treatment of SARS-CoV-2 infection (Magagnoli et al., 2020).

Corticosteroids are the most conventional immunosuppressant drugs used to suppress inflammatory responses (Cinatl et al., 2005). Although the WHO cautions of their use, they have been widely used despite lack of scientific data. Furthermore, because of the high incidence of arterial hypertension, diabetes, and congestive heart failure in subjects with COVID-19, corticosteroids should be used with caution. It is well-described that corticosteroids potentiate the effect of Ang II and RAS (Ullian et al., 1996), hence it is less likely that corticosteroids provide any significant clinical benefit in this clinical scenario.

### Manipulation of ACE2/Ang(1-7) and Protease Activity as Novel Therapeutic Targets

Considering the significant SARS-CoV-2 related risk factors for hospitalization and mortality among patients with metabolic diseases, including obesity, arterial hypertension, cardiovascular diseases, and diabetes that may reflect overall activation of the RAS system, modulation of RAS activation through the ACE2/(Ang1-7)/MAS pathway should be considered for treatment of this disease. Furthermore, our clinical observation and published clinical data suggest a unique clinical presentation of SARS-CoV-2 patients: most patients present with relatively preserved hemodynamics and lack of lactic acidosis. But they have respiratory distress, appear to be in a hypercoagulable state (Liu et al., 2020; Menter et al., 2020), exhibit progressive renal failure (Cheng et al., 2020), have stroke like features and myocardial injury (Zhou et al., 2020). Clinical observational studies indicate that in most cases the respiratory distress occurs many days (in general about 14 days) after the infection, suggesting that this may not be a direct effect of the initial viral infection but rather the hosts reaction to the loss of function of ACE2 and dysregulation of Ang II/ACE2 pathways as well activation of host proteases. Our central hypothesis is that the binding of the coronavirus spike protein to ACE2 leads to shedding of ACE2 receptors by various proteases, which in turn leads to the loss of protective function of the ACE2/MAS axis in the lungs and other organs (Figure 1B). In addition to the loss of protective function of ACE2/MAS, activation of classical pathway (ACE/RAS/Ang II) and alternative pathways through tissue specific proteases, including cathepsins, chymase-like proteases, leads to an excessive production of Ang II at the tissue level. This process may further shift the balance of protective Ang (1-7)/MAS and ACE2 function to the detrimental effects of increased Ang II contributing to lung epithelial and endovascular injury. Therefore, induction of the downstream pathway of ACE2, by activating the ACE2/Ang1-7/MAS axis may prove a useful strategy in preventing lung and cardiovascular damage associated with SARS-CoV-2 infections. Because decreased ACE2/MAS activity augments the Ang II/AT1R activity and its hazardous consequence on increased pulmonary vascular endothelial/epithelial injury and lung pathology. Inhibiting the activity of proteases necessary for cleavage of viral spike proteins: for instance inhibition of enzymatic activity of ADAM17 and TMPRSS2 could serve as other novel therapeutic targets. This could potentially block viral interaction with the receptor and its entry into the cells. Identification of specific proteases and development of inhibitors targeting proteases necessary for cleavage of spike proteins may prove to be viable. In addition, exploiting the protective effect of Ang1-7 or its analogs, such as AVE0991 AVE0991 (Pinheiro et al., 2004) against deleterious effect of increased Ang II is feasible and might be effective for the symptomatic treatment of these patients.

## Concluding Remarks

Based on the importance of ACE2 as a counterbalance to the deleterious effects of Ang II, the loss of ACE2 and Ang(1-7) may be detrimental to the organism. Surprisingly, little is known about the effect of SARS-CoV-2 virus binding to ACE2 and how the viral binding on this receptor may modulate the ACE2 enzymatic activity impact its role as a “survival factor.” Critical questions that are yet to be answered include: (1) What effect does SARS-CoV-2 binding to ACE2 have on its enzymatic activity, and on its protective actions toward lung epithelial cells and lung injury? (2) What effect(s) does SARS-CoV-2 infection of lung epithelial cells/endothelial cells have on ACE2 expression in the lungs and other organs? (3) Do known inhibitors or activators of ACE2 have any effect(s) on the binding of SARS-CoV-2 to the ACE2 receptor and/or infection of lung epithelial cells? Regardless, these are questions of fundamental importance to our understanding of SARS-CoV-2 biology that need to be answered soon.

## Data Availability Statement

The original contributions presented in the study are included in the article/supplementary material, further inquiries can be directed to the corresponding authors.

## Author Contributions

Both authors read and approved the final manuscript.

## Conflict of Interest

The authors declare that the research was conducted in the absence of any commercial or financial relationships that could be construed as a potential conflict of interest.

## References

[B1] Assiri A. Al-Tawfiq J. A. Al-Rabeeah A. A. Al-Rabiah F. A. Al-Hajjar S. Al-Barrak A.. (2013). Epidemiological, demographic, and clinical characteristics of 47 cases of Middle East respiratory syndrome coronavirus disease from Saudi Arabia: a descriptive study. Lancet Infect Dis. 13, 752–761. 10.1016/S1473-3099(13)70204-4 23891402 PMC7185445

[B2] Baud D. Qi X. Nielsen-Saines K. Musso D. Pomar L. Favre G. (2020). Real estimates of mortality following COVID-19 infection. Lancet Infect. Dis. 10.1016/S1473-3099(20)30195-X. [Epub ahead of print]. 32171390 PMC7118515

[B3] Becari C. Oliveira E. B. Salgado M. C. O. (2011). Alternative pathways for angiotensin II generation in the cardiovascular system. Braz. J. Med. Biol. Res. 44, 914–919. 10.1590/S0100-879X2011007500093 21956534

[B4] Belouzard S. Chu V. C. Whittaker G. R. (2009). Activation of the SARS coronavirus spike protein via sequential proteolytic cleavage at two distinct sites. Proc. Natl. Acad. Sci. U.S.A. 106, 5871–5876. 10.1073/pnas.0809524106 19321428 PMC2660061

[B5] Cao B. Wang Y. Wen D. Liu W. Wang J. Fan G.. (2020). A trial of lopinavir–ritonavir in adults hospitalized with severe Covid-19. N. Engl. J. Med. 7, 1787–1799. 10.1056/NEJMoa2001282 32187464 PMC7121492

[B6] Cerdà-Costa N. Xavier Gomis-Rüth F. (2014). Architecture and function of metallopeptidase catalytic domains. Protein Sci. 23, 123–144. 10.1002/pro.2400 24596965 PMC3926739

[B7] Cheng Y. Luo R. Wang K. Zhang M. Wang Z. Dong L.. (2020). Kidney disease is associated with in-hospital death of patients with COVID-19. Kidney Int. 97, 829–838. 10.1016/j.kint.2020.03.005 32247631 PMC7110296

[B8] Chu C.-M. Poon L. L. Cheng V. C. Chan K.-S. Hung I. F. Wong M. M.. (2004). Initial viral load and the outcomes of SARS. CMAJ 171, 1349–1352. 10.1503/cmaj.1040398 15557587 PMC527336

[B9] Cinatl J. Michaelis M. Morgenstern B. Doerr H. W. (2005). High-dose hydrocortisone reduces expression of the pro-inflammatory chemokines CXCL8 and CXCL10 in SARS coronavirus-infected intestinal cells. Int. J. Mol. Med. 15, 323–327. 10.3892/ijmm.15.2.323 15647850

[B10] Crackower M. A. Sarao R. Oudit G. Y. Yagil C. Kozieradzki I. Scanga S. E.. (2002). Angiotensin-converting enzyme 2 is an essential regulator of heart function. Nature 417, 822–828. 10.1038/nature00786 12075344

[B11] de Kloet A. D. Krause E. G. Woods S. C. (2010). The renin angiotensin system and the metabolic syndrome. Physiol. Behav. 100, 525–534. 10.1016/j.physbeh.2010.03.018 20381510 PMC2886177

[B12] Diaz J. H. (2020). Hypothesis: angiotensin-converting enzyme inhibitors and angiotensin receptor blockers may increase the risk of severe COVID-19. J Travel Med. 27:taaa041. 10.1093/jtm/taaa041 32186711 PMC7184445

[B13] Donoghue M. Hsieh F. Baronas E. Godbout K. Gosselin M. Stagliano N.. (2000). A novel angiotensin-converting enzyme–related carboxypeptidase (ACE2) converts angiotensin I to angiotensin 1-9. Circul. Res. 87, e1–e9. 10.1161/01.RES.87.5.e1 10969042

[B14] Fehr A. R. Perlman S. (2015). Coronaviruses: an overview of their replication and pathogenesis. Coronaviruses 1282, 1–23. 10.1007/978-1-4939-2438-7_1 25720466 PMC4369385

[B15] Fouchier R. A. Hartwig N. G. Bestebroer T. M. Niemeyer B. de Jong J. C. Simon J. H.. (2004). A previously undescribed coronavirus associated with respiratory disease in humans. Proc. Natl. Acad. Sci. U.S.A. 101, 6212–6216. 10.1073/pnas.0400762101 15073334 PMC395948

[B16] Fraga-Silva R. A. Sorg B. S. Wankhede M. deDeugd C. Jun J. Y. Baker M. B.. (2010). ACE2 activation promotes antithrombotic activity. Mol. Med. 16, 210–215. 10.2119/molmed.2009.00160 20111697 PMC2811560

[B17] Funakoshi Y. Ichiki T. Ito K. Takeshita A. (1999). Induction of interleukin-6 expression by angiotensin II in rat vascular smooth muscle cells. Hypertension 34, 118–125. 10.1161/01.HYP.34.1.118 10406834

[B18] Garrido A. M. Griendling K. K. (2009). NADPH oxidases and angiotensin II receptor signaling. Mol. Cell. Endocrinol. 302, 148–158. 10.1016/j.mce.2008.11.003 19059306 PMC2835147

[B19] Glass W. G. Subbarao K. Murphy B. Murphy P. M. (2004). Mechanisms of host defense following severe acute respiratory syndrome-coronavirus (SARS-CoV) pulmonary infection of mice. J. Immunol. 173, 4030–4039. 10.4049/jimmunol.173.6.4030 15356152

[B20] Gopallawa I. Uhal B. D. (2014). Molecular and cellular mechanisms of the inhibitory effects of ACE-2/ANG1-7/Mas axis on lung injury. Curr. Top. Pharmacol. 18:71. 26146467 PMC4487538

[B21] Gopallawa I. Uhal B. D. (2016). Translational research in acute lung injury and pulmonary fibrosis: angiotensin-(1-7)/mas inhibits apoptosis in alveolar epithelial cells through upregulation of MAP kinase phosphatase-2. Am. J. Physiol. Lung Cell. Mol. Physiol. 310:L240 10.1152/ajplung.00187.2015 26637635 PMC4888557

[B22] Grasselli G. Pesenti A. Cecconi M. (2020). Critical care utilization for the COVID-19 outbreak in Lombardy, Italy: early experience and forecast during an emergency response. 333, 1545–1546 JAMA. 10.1001/jama.2020.4031 32167538

[B23] Guo Y.-R. Cao Q.-D. Hong Z.-S. Tan Y.-Y. Chen S.-D. Jin H.-J.. (2020). The origin, transmission and clinical therapies on coronavirus disease 2019 (COVID-19) outbreak–an update on the status. Military Med. Res. 7, 1–10. 10.1186/s40779-020-00240-0 32169119 PMC7068984

[B24] Gustafsson F. Holstein-Rathlou N.-H. (1999). Angiotensin II modulates conducted vasoconstriction to norepinephrine and local electrical stimulation in rat mesenteric arterioles. Cardiovasc. Res. 44, 176–184. 10.1016/S0008-6363(99)00174-1 10615401

[B25] Hamming I. Timens W. Bulthuis M. Lely A. Navis G. van Goor H. (2004). Tissue distribution of ACE2 protein, the functional receptor for SARS coronavirus. A first step in understanding SARS pathogenesis. J. Pathol. 203, 631–637. 10.1002/path.1570 15141377 PMC7167720

[B26] Han Y. Runge M. S. Brasier A. R. (1999). Angiotensin II induces interleukin-6 transcription in vascular smooth muscle cells through pleiotropic activation of nuclear factor-κB transcription factors. Circul. Res. 84, 695–703. 10.1161/01.RES.84.6.695 10189357

[B27] Heurich A. Hofmann-Winkler H. Gierer S. Liepold T. Jahn O. Pöhlmann S. (2014). TMPRSS2 and ADAM17 cleave ACE2 differentially and only proteolysis by TMPRSS2 augments entry driven by the severe acute respiratory syndrome coronavirus spike protein. J. Virol. 88, 1293–1307. 10.1128/JVI.02202-13 24227843 PMC3911672

[B28] Hoffmann M. Kleine-Weber H. Schroeder S. Krüger N. Herrler T. Erichsen S.. (2020). SARS-CoV-2 cell entry depends on ACE2 and TMPRSS2 and is blocked by a clinically proven protease inhibitor. Cell. 181, 271–280. 10.1016/j.cell.2020.02.052 32142651 PMC7102627

[B29] Huang C. Wang Y. Li X. Ren L. Zhao J. Hu Y.. (2020). Clinical features of patients infected with 2019 novel coronavirus in Wuhan, China. Lancet 395, 497–506. 10.1016/S0140-6736(20)30183-5 31986264 PMC7159299

[B30] Ishiyama Y. Gallagher P. E. Averill D. B. Tallant E. A. Brosnihan K. B. Ferrario C. M. (2004). Upregulation of angiotensin-converting enzyme 2 after myocardial infarction by blockade of angiotensin II receptors. Hypertension 43, 970–976. 10.1161/01.HYP.0000124667.34652.1a 15007027

[B31] Jagroop I. Mikhailidis D. (2000). Angiotensin II can induce and potentiate shape change in human platelets: effect of losartan. J. Hum. Hypertens. 14, 581–585. 10.1038/sj.jhh.1001102 10980590

[B32] Jang Y. Kim S. M. (2012). Influences of the G2350A polymorphism in the ACE Gene on cardiac structure and function of ball game players. J. Negative Results Biomed. 11:6. 10.1186/1477-5751-11-6 22239999 PMC3278340

[B33] Jiang T. Gao L. Lu J. Zhang Y.-D. (2013). ACE2-Ang-(1-7)-Mas axis in brain: a potential target for prevention and treatment of ischemic stroke. Curr. Neuropharmacol. 11, 209–217. 10.2174/1570159X11311020007 23997755 PMC3637674

[B34] Kato H. Hou J. Chobanian A. V. Brecher P. (1996). Effects of angiotensin II infusion and inhibition of nitric oxide synthase on the rat aorta. Hypertension 28, 153–158. 10.1161/01.HYP.28.2.153 8707375

[B35] Kirchdoerfer R. N. Cottrell C. A. Wang N. Pallesen J. Yassine H. M. Turner H. L.. (2016). Pre-fusion structure of a human coronavirus spike protein. Nature 531, 118–121. 10.1038/nature17200 26935699 PMC4860016

[B36] Kramkowski K. Mogielnicki A. Buczko W. (2006). The physiological significance of the alternative. J. Physiol. Pharmacol. 57, 529–539. 17229979

[B37] Krijnse-Locker J. Ericsson M. Rottier P. Griffiths G. (1994). Characterization of the budding compartment of mouse hepatitis virus: evidence that transport from the RER to the Golgi complex requires only one vesicular transport step. J. Cell Biol. 124, 55–70. 10.1083/jcb.124.1.55 8294506 PMC2119890

[B38] Lambert D. W. Yarski M. Warner F. J. Thornhill P. Parkin E. T. Smith A. I.. (2005). Tumor necrosis factor-α convertase (ADAM17) mediates regulated ectodomain shedding of the severe-acute respiratory syndrome-coronavirus (SARS-CoV) receptor, angiotensin-converting enzyme-2 (ACE2). J. Biol. Chem. 280, 30113–30119. 10.1074/jbc.M505111200 15983030 PMC8062222

[B39] le Tran Y. Forster C. (1997). Angiotensin-(1-7) and the rat aorta: modulation by the endothelium. J. Cardiovasc. Pharmacol. 30, 676–682. 10.1097/00005344-199711000-00019 9388051

[B40] Li F. Li W. Farzan M. Harrison S. C. (2005). Structure of SARS coronavirus spike receptor-binding domain complexed with receptor. Science 309, 1864–1868. 10.1126/science.1116480 16166518

[B41] Li W. Moore M. J. Vasilieva N. Sui J. Wong S. K. Berne M. A.. (2003). Angiotensin-converting enzyme 2 is a functional receptor for the SARS coronavirus. Nature 426, 450–454. 10.1038/nature02145 14647384 PMC7095016

[B42] Li X. Molina-Molina M. Abdul-Hafez A. Uhal V. Xaubet A. Uhal B. D. (2008). Angiotensin converting enzyme-2 is protective but downregulated in human and experimental lung fibrosis. Am. J. Physiol. Lung Cell. Mol. Physiol. 295, L178–L185. 10.1152/ajplung.00009.2008 18441099 PMC2494775

[B43] Liu X. Li Z. Liu S. Chen Z. Zhao Z. Huang Y.-y. (2020). Therapeutic effects of dipyridamole on COVID-19 patients with coagulation dysfunction. medRxiv. 10.1101/2020.02.27.20027557

[B44] Lorenz J. N. (2010). Chymase: The Other ACE? Bethesda, MD: American Physiological Society.

[B45] Lu H. Ai J. Shen Y. Li Y. Li T. Zhou X. (2020). A descriptive study of the impact of diseases control and prevention on the epidemics dynamics and clinical features of SARS-CoV-2 outbreak in Shanghai, lessons learned for metropolis epidemics prevention. medRxiv. 10.1101/2020.02.19.20025031

[B46] Luther J. M. Gainer J. V. Murphey L. J. Yu C. Vaughan D. E. Morrow J. D.. (2006). Angiotensin II induces interleukin-6 in humans through a mineralocorticoid receptor–dependent mechanism. Hypertension 48, 1050–1057. 10.1161/01.HYP.0000248135.97380.76 17043157

[B47] Magagnoli J. Narendran S. Pereira F. Cummings T. Hardin J. W. Sutton S. S. (2020). Outcomes of hydroxychloroquine usage in United States veterans hospitalized with Covid-19. medRxiv. 10.1101/2020.04.16.20065920 PMC7274588 32838355

[B48] Malhotra A. Kang B. P. Cheung S. Opawumi D. Meggs L. G. (2001). Angiotensin II promotes glucose-induced activation of cardiac protein kinase C isozymes and phosphorylation of troponin I. Diabetes 50, 1918–1926. 10.2337/diabetes.50.8.1918 11473056

[B49] Marian A. (2013). The discovery of the ACE2 gene. Circul. Res. 112, 1307–1309. 10.1161/CIRCRESAHA.113.301271 23661710

[B50] Martin R. E. Marchetti R. V. Cowan A. I. Howitt S. M. Bröer S. Kirk K. (2009). Chloroquine transport via the malaria parasite's chloroquine resistance transporter. Science 325, 1680–1682. 10.1126/science.1175667 19779197

[B51] Menter T. Haslbauer J. D. Nienhold R. Savic S. Hopfer H. Deigendesch N.. (2020). Post-mortem examination of COVID19 patients reveals diffuse alveolar damage with severe capillary congestion and variegated findings of lungs and other organs suggesting vascular dysfunction. Histopathology. 10.1111/his.14134. [Epub ahead of print]. 32364264 PMC7496150

[B52] Mento P. F. Wilkes B. M. (1987). Plasma angiotensins and blood pressure during converting enzyme inhibition. Hypertension 9:III42. 10.1161/01.HYP.9.6_Pt_2.III42 3036705

[B53] Moore J. X. Chaudhary N. Akinyemiju T. (2017). Peer reviewed: metabolic syndrome prevalence by race/ethnicity and sex in the United States, National Health and Nutrition Examination Survey, 1988–2012. Prev. Chronic Dis. 14:E24. 10.5888/pcd14.160287 28301314 PMC5364735

[B54] Mulangu S. Dodd L. E. Davey R. T. Jr Tshiani Mbaya O. Proschan M. Mukadi D.. (2019). A randomized, controlled trial of Ebola virus disease therapeutics. N. Engl. J. Med. 381, 2293–2303. 10.1056/NEJMoa1910993 31774950 PMC10680050

[B55] Nabah Y. N. A. Mateo T. Estells R. Mata M. Zagorski J. Sarau H.. (2004). Angiotensin II induces neutrophil accumulation *in vivo* through generation and release of CXC chemokines. Circulation 110, 3581–3586. 10.1161/01.CIR.0000148824.93600.F3 15569833

[B56] Nguyen H. Uhal B. D. (2016). The unfolded protein response controls ER stress-induced apoptosis of lung epithelial cells through angiotensin generation. Am. J. Physiol. Lung Cell. Mol. Physiol. 311, L846–L854. 10.1152/ajplung.00449.2015 27638906 PMC5130534

[B57] Oarhe C. I. Dang V. Dang M. Nguyen H. Gopallawa I. Gewolb I. H.. (2015). Hyperoxia downregulates angiotensin-converting enzyme-2 in human fetal lung fibroblasts. Pediatric Res. 77, 656–662. 10.1038/pr.2015.27 25665060 PMC5119454

[B58] Oudit G. Kassiri Z. Jiang C. Liu P. Poutanen S. Penninger J.. (2009). SARS-coronavirus modulation of myocardial ACE2 expression and inflammation in patients with SARS. Eur. J. Clin. Invest. 39, 618–625. 10.1111/j.1365-2362.2009.02153.x 19453650 PMC7163766

[B59] Pinheiro S. V. B. Simoes e Silva A. C. Sampaio W. O. de Paula R. D. Mendes E. P. Bontempo E. D.. (2004). Nonpeptide AVE 0991 is an angiotensin-(1-7) receptor Mas agonist in the mouse kidney. Hypertension 44, 490–496. 10.1161/01.HYP.0000141438.64887.42 15326087

[B60] Porcheddu R. Serra C. Kelvin D. Kelvin N. Rubino S. (2020). Similarity in Case Fatality Rates (CFR) of COVID-19/SARS-COV-2 in Italy and China. J. Infect. Dev. Ctries 14, 125–128. 10.3855/jidc.12600 32146445

[B61] Raj V. S. Mou H. Smits S. L. Dekkers D. H. Müller M. A. Dijkman R.. (2013). Dipeptidyl peptidase 4 is a functional receptor for the emerging human coronavirus-EMC. Nature 495, 251–254. 10.1038/nature12005 23486063 PMC7095326

[B62] Ramachandran V. Ismail P. Stanslas J. Shamsudin N. Moin S. Mohd Jas R. (2008). Association of insertion/deletion polymorphism of angiotensin-converting enzyme gene with essential hypertension and type 2 diabetes mellitus in Malaysian subjects. J. Renin Angiotensin Aldosterone Syst. 9, 208–214. 10.1177/1470320308097499 19126661

[B63] Rey-Parra G. Vadivel A. Coltan L. Hall A. Eaton F. Schuster M.. (2012). Angiotensin converting enzyme 2 abrogates bleomycin-induced lung injury. J. Mol. Med. 90, 637–647. 10.1007/s00109-012-0859-2 22246130 PMC7080102

[B64] Roca-Ho H. Riera M. Palau V. Pascual J. Soler M. J. (2017). Characterization of ACE and ACE2 expression within different organs of the NOD mouse. Int. J. Mol. Sci. 18:563. 10.3390/ijms18030563 28273875 PMC5372579

[B65] Ruiz-Ortega M. Ruperez M. Lorenzo O. Esteban V. Blanco J. Mezzano S.. (2002). Angiotensin II regulates the synthesis of proinflammatory cytokines and chemokines in the kidney. Kidney Int. 62, S12–S22. 10.1046/j.1523-1755.62.s82.4.x 12410849

[B66] Santos R. A. e Silva A. C. S. Maric C. Silva D. M. Machado R. P. de Buhr I. (2003). Angiotensin-(1-7) is an endogenous ligand for the G protein-coupled receptor Mas. Proc. Natl. Acad. Sci. U.S.A. 100, 8258–8263. 10.1073/pnas.1432869100 12829792 PMC166216

[B67] Schindler C. Bramlage P. Kirch W. Ferrario C. M. (2007). Role of the vasodilator peptide angiotensin-(1-7) in cardiovascular drug therapy. Vasc. Health Risk Manage. 3:125. 17583183 PMC1994039

[B68] Senchenkova E. Y. Russell J. Almeida-Paula L. D. Harding J. W. Granger D. N. (2010). Angiotensin II–mediated microvascular thrombosis. Hypertension 56, 1089–1095. 10.1161/HYPERTENSIONAHA.110.158220 20975035 PMC3023299

[B69] Simoes e Silva A. Silveira K. Ferreira A. Teixeira M. (2013). ACE2, angiotensin-(1-7) and M as receptor axis in inflammation and fibrosis. Br. J. Pharmacol. 169, 477–492. 10.1111/bph.12159 23488800 PMC3682698

[B70] Singh K. D. Karnik S. S. (2016). Angiotensin receptors: structure, function, signaling and clinical applications. J. Cell Signal. 1:111. 10.4172/jcs.1000111 27512731 PMC4976824

[B71] Sohrabi C. Alsafi Z. O'Neill N. Khan M. Kerwan A. Al-Jabir A.. (2020). World Health Organization declares global emergency: a review of the 2019 novel coronavirus (COVID-19). Int. J. Surg. 76, 71–76. 10.1016/j.ijsu.2020.02.034 32112977 PMC7105032

[B72] Talreja J. Talwar H. Bauerfeld C. Grossman L. I. Zhang K. Tranchida P.. (2019). HIF-1alpha regulates IL-1beta and IL-17 in sarcoidosis. Elife 8:e44519. 10.7554/eLife.44519.026 30946009 PMC6506207

[B73] Tay K.-H. Lip G. Y. (2008). What “Drives” the Link Between the Renin–Angiotensin–Aldosterone System and the Prothrombotic State in Hypertension? Oxford, UK: Oxford University Press. 10.1038/ajh.2008.315 19020509

[B74] Tikellis C. Thomas M. (2012). Angiotensin-converting enzyme 2 (ACE2) is a key modulator of the renin angiotensin system in health and disease. Int. J. Peptides 2012:256294. 10.1155/2012/256294 22536270 PMC3321295

[B75] Turner A. J. Tipnis S. R. Guy J. L. Rice G. I. Hooper N. M. (2002). ACEH/ACE2 is a novel mammalian metallocarboxypeptidase and a homologue of angiotensin-converting enzyme insensitive to ACE inhibitors. Can. J. Physiol. Pharmacol. 80, 346–353. 10.1139/y02-021 12025971

[B76] Uehara Y. Miura S.-i. Yahiro E. Saku K. (2013). Non-ACE pathway-induced angiotensin II production. Curr. Pharm. Des. 19, 3054–3059. 10.2174/1381612811319170012 23176219

[B77] Uhal B. D. Li X. Xue A. Gao X. Abdul-Hafez A. (2011). Regulation of alveolar epithelial cell survival by the ACE-2/angiotensin 1-7/Mas axis. Am. J. Physiol. Lung Cell. Mol. Physiol. 301, L269–L274. 10.1152/ajplung.00222.2010 21665960 PMC3174737

[B78] Ullian M. E. Walsh L. G. Morinelli T. A. (1996). Potentiation of angiotensin II action by corticosteroids in vascular tissue. Cardiovasc. Res. 32, 266–273. 10.1016/0008-6363(96)00053-3 8796113

[B79] Wakahara S. Konoshita T. Mizuno S. Motomura M. Aoyama C. Makino Y.. (2007). Synergistic expression of angiotensin-converting enzyme (ACE) and ACE2 in human renal tissue and confounding effects of hypertension on the ACE to ACE2 ratio. Endocrinology 148, 2453–2457. 10.1210/en.2006-1287 17303661

[B80] Wang M. Cao R. Zhang L. Yang X. Liu J. Xu M.. (2020). Remdesivir and chloroquine effectively inhibit the recently emerged novel coronavirus (2019-nCoV) *in vitro*. Cell Res. 30, 269–271. 10.1038/s41422-020-0282-0 32020029 PMC7054408

[B81] Wang Q. Zhang Y. Wu L. Niu S. Song C. Zhang Z.. (2020). Structural and functional basis of SARS-CoV-2 entry by using human ACE2. Cell 181, 894–904.e9. 10.1016/j.cell.2020.03.045 32275855 PMC7144619

[B82] Wang R. Zagariya A. Ibarra-Sunga O. Gidea C. Ang E. Deshmukh S.. (1999). Angiotensin II induces apoptosis in human and rat alveolar epithelial cells. Am. J. Physiol. Lung Cell. Mol. Physiol. 276, L885–L889. 10.1152/ajplung.1999.276.5.L885 10330045

[B83] Watanabe T. Barker T. A. Berk B. C. (2005). Angiotensin II and the endothelium: diverse signals and effects. Hypertension 45, 163–169. 10.1161/01.HYP.0000153321.13792.b9 15630047

[B84] World Health Organization (2013). Revised Interim Case Definition for Reporting to WHO-Middle East Respiratory Syndrome Coronavirus (MERS-CoV) (Geneva).

[B85] Xue B. Beltz T. G. Yu Y. Guo F. Gomez-Sanchez C. E. Hay M.. (2011). Central interactions of aldosterone and angiotensin II in aldosterone-and angiotensin II-induced hypertension. Am. J. Physiol. Heart Circul. Physiol. 300, H555–H564. 10.1152/ajpheart.00847.2010 21112947 PMC3044048

[B86] Yamamoto K. Ohishi M. Katsuya T. Ito N. Ikushima M. Kaibe M.. (2006). Deletion of angiotensin-converting enzyme 2 accelerates pressure overload-induced cardiac dysfunction by increasing local angiotensin II. Hypertension 47, 718–726. 10.1161/01.HYP.0000205833.89478.5b 16505206

[B87] Yan R. Zhang Y. Li Y. Xia L. Guo Y. Zhou Q. (2020). Structural basis for the recognition of SARS-CoV-2 by full-length human ACE2. Science 367, 1444–1448. 10.1126/science.abb2762 32132184 PMC7164635

[B88] Zhou F. Yu T. Du R. Fan G. Liu Y. Liu Z.. (2020). Clinical course and risk factors for mortality of adult inpatients with COVID-19 in Wuhan, China: a retrospective cohort study. Lancet. 395, 1054–1062. 10.1016/S0140-6736(20)30566-3 32171076 PMC7270627

[B89] Zhu L. She Z. G. Cheng X. Qin J. J. Zhang X. J. Cai J.. (2020). Association of blood glucose control and outcomes in patients with COVID-19 and pre-existing type 2 diabetes. Cell Metab. 10.1016/j.cmet.2020.04.021. [Epub ahead of print]. 32369736 PMC7252168

